# Treatment Outcomes of Upside–Down Descemet Membrane Endothelial Keratoplasty

**DOI:** 10.3390/jcm14186427

**Published:** 2025-09-12

**Authors:** Karolina Bonińska, Sławomir Cisiecki, Tomasz Dybek, Agnieszka Kardaszewska, Maciej Bednarski

**Affiliations:** 1 Centrum Medyczne “Julianów”, ul. Żeglarska 4, 91-321 Łódź, Poland; 2 Miejskie Centrum Medyczne JONSCHER, ul. Milionowa 14, 93-113 Łódź, Poland; 3 Prof. Zagórski Eye Surgery Centers, OCHO Medical Group, 31-216 Kraków, Poland

**Keywords:** Descemet membrane endothelial keratoplasty, endothelial keratoplasty, Fuchs’ endothelial corneal dystrophy, intraoperative-OCT, upside–down implantation

## Abstract

**Background**: To present the management of upside–down Descemet membrane endothelial keratoplasty (DMEK) with intraoperative optical coherence tomography (iOCT). **Methods**: We report the case of a 74-year-old woman who underwent DMEK for Fuchs’ endothelial corneal dystrophy (FECD) of the right eye. Preoperative corrected distance visual acuity (CDVA) was 20/1500 (counting fingers [CF]), and the central corneal thickness (CCT) was 637 μm. No graft markings were made. Graft positioning was determined using iOCT. An improper graft position was suspected due to the lack of postoperative anatomical and functional improvements. The CDVA noted at that time was 20/1500 (CF), and the CCT was 708 μm. The graft was subsequently repositioned. This procedure was performed 33 days after the primary surgery. **Results**: The central corneal thickness and CDVA were 495 μm and 20/40 sc, respectively, at the final 18-month postoperative follow-up. **Conclusions**: Incorrect positioning of the transplanted corneal graft should be considered if no improvement is observed after DMEK. The learning curve significantly affects the occurrence of these complications. Graft repositioning, even 33 days after surgery, is associated with a favorable prognosis.

## 1. Introduction

Descemet membrane endothelial keratoplasty (DMEK) is a partial thickness corneal transplantation. It is currently the gold standard surgical treatment for corneal endothelial disorders [1,2]. In contrast to earlier methods such as penetrating keratoplasty (PK) or Descemet stripping endothelial keratoplasty (DSEK/DSAEK), DMEK involves the transplantation of only the Descemet membrane (DM) and the endothelial layer from a donor cornea [1,2]. It excludes the donor stroma from the graft, thus resulting in a shorter postoperative recovery time, lower rejection rate, superior visual outcomes, and reduced postoperative astigmatism than earlier techniques [1,2].

The primary indications for DMEK include Fuchs’ endothelial corneal dystrophy (FECD) and pseudophakic bullous keratopathy [2]. FECD is the most prevalent condition, especially in developed countries, affecting up to 4% of individuals aged > 40 years [3]. With the aging of the global population, the burden of endothelial disease is expected to increase, leading to a demand for advanced corneal transplantation techniques. Therefore, DMEK has become the preferred surgical approach for several patients [4].

The advantages of DMEK are well known. The absence of donor stroma reduces interface haze, allowing near-anatomical corneal restoration. Postoperative refractive stability is markedly improved with minimal surgically induced astigmatism. In addition, the risk of graft rejection is significantly lower than that with PK, with certain studies reporting rejection rates below 1% [5]. The smaller graft volume and absence of sutures further reduce inflammation and contribute to the favorable safety profile of the procedure.

Despite these advantages, DMEK is technically demanding. The graft itself is very thin, typically 10–20 μm, making it fragile and difficult to handle intraoperatively. Successful outcomes depend on the tissue preparation, careful manipulation, and precise orientation of the graft. One of the most critical intraoperative challenges is ensuring the correct positioning of the Descemet–endothelium complex. If the graft is inadvertently implanted upside down, the endothelial cells face the anterior chamber rather than the stromal surface, inevitably causing primary graft failure due to lack of endothelial function [6].

Several strategies have been proposed to minimize the risk of misorientation, including peripheral graft marking, intraoperative optical coherence tomography (iOCT) use, and graft’s natural scrolling behavior assessment, which typically rolls the endothelium out [7,8]. Other intraoperative signs have also been described, such as the double-line reflection pattern, which provides a simple method to confirm graft orientation during DMEK [9]. Each method has advantages and limitations. Although stamping provides a proper reference, it may cause endothelial damage. Similarly, iOCT is noninvasive and real-time; it requires both high-resolution imaging and surgical experience, and it may still be inconclusive in certain settings.

Here, we present a case of upside–down DMEK graft implantation that was identified postoperatively and successfully corrected 33 days after the initial surgery. This case highlights both the promise and limitations of iOCT-guided DMEK and contributes to the ongoing discussion on improving surgical outcomes and graft survival. To the best of our knowledge, only a few reports have described delayed graft repositioning with subsequent clinical success, making this case a valuable addition to the literature.

## 2. Case Report

**Figure 1:**
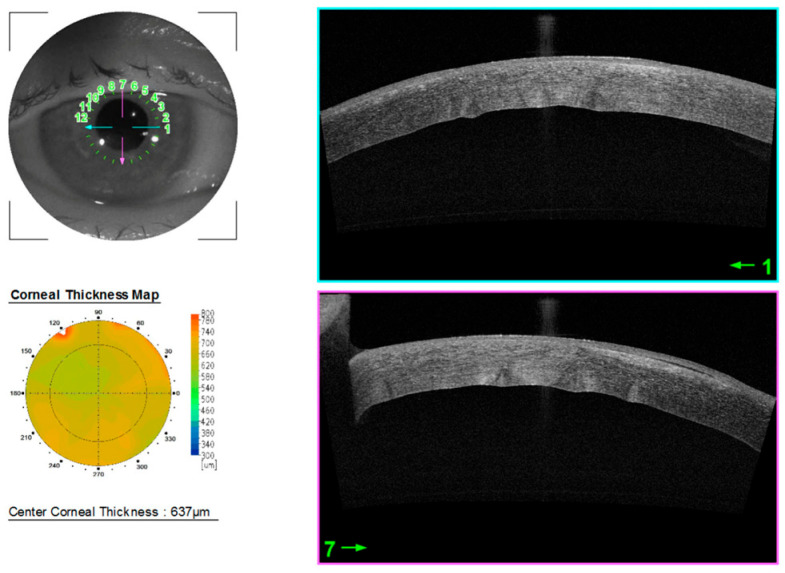
Anterior segment OCT before surgery.

A 74-year-old woman was admitted to the ophthalmology department with progressive keratopathy of the right eye secondary to FECD, scheduled for DMEK. The patient was pseudophakic. The corrected distance visual acuity (CDVA) values in the right and left eyes were 20/1500 and 20/20, respectively. Intraocular pressure in both eyes was within the normal range. SS-OCT (3D DRI OCT Triton) revealed the presence of an epiretinal membrane in the macular region of both eyes. Anterior segment optical coherence tomography (AS-OCT, 3D DRI OCT Triton) revealed a central corneal thickness (CCT) of 637 μm in the right eye (Figure 1). The average endothelial cell density (CD) of the donor tissue from a 39-year-old woman was 2611 cells/mm^2^ (TOMEY Specular microscope EM-4000, Phoenix, AZ, USA). The cornea came from the Eye Tissue Bank in Warsaw. Eusol-C was used for cold corneal storage. The procedure was performed electively under local peribulbar anesthesia by a certified operator (S.C.).

### Surgical Technique

Surgery begins with the preparation of the donor tissue using a liquid bubble technique [10]. After identifying the iris base, sharp dissection was performed with a standard paracentesis knife underneath the iris base into Schlemm’s canal. The preparation was continued using a blunt spatula (Geuder; Heidelberg, Germany). Once the area with more resistance has been overcome, the DM can be easily separated from the stromal layer to create a sub-Descemet tunnel with a 2 mm width and 3 mm length [10]. A blunt cannula was introduced into the sub-Descemet pocket, and the prepared tunnel was manually closed by applying pressure using a small surgical pad to prevent reflux during injection. Trypan blue (DORC, Zuidland, The Netherlands) was injected into the sub-Descemet space to form an enlarging liquid bubble, creating a complete detachment of the DM. After corneal trephination with an 8.25 mm trephine, the donor cornea was retained in the storage solution [10].

A clear corneal incision and four paracenteses were made after application of 5% povidone–iodine for two minutes. Peripheral iridectomy was performed in the 6 o’clock position, followed by descemetorhexis under balanced salt solution (BSS) irrigation. The presence of residual DM was confirmed by applying membrane blue (DORC), and remnants of the DM within the 8.25 mm descemetorhexis area were subsequently removed. Under fluid irrigation, the graft was introduced into the anterior chamber using a 2.6 mm clear corneal incision. The orientation of the DMEK graft was confirmed using intraoperative OCT (Leica Proveo 8). Subsequently, a 10% sulfur hexafluoride (SF_6_) gas tamponade was used.

A follow-up OCT performed the day after surgery revealed a reduction in CCT to 573 μm. The patient was then discharged and recommended for further follow-up.

**Figure 2:**
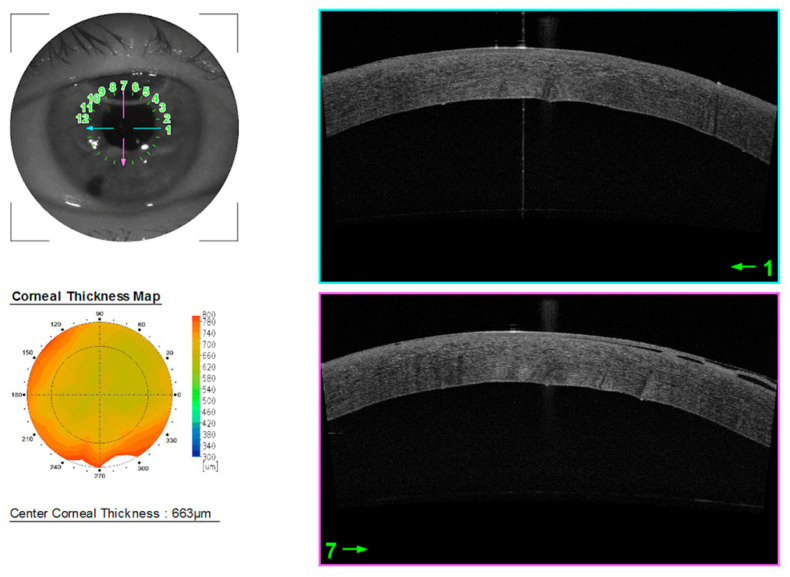
Anterior segment OCT 6 days after surgery.

Six days after the surgery, CDVA did not improve (20/1500), whereas CCT was 663 μm (Figure 2).

**Figure 3:**
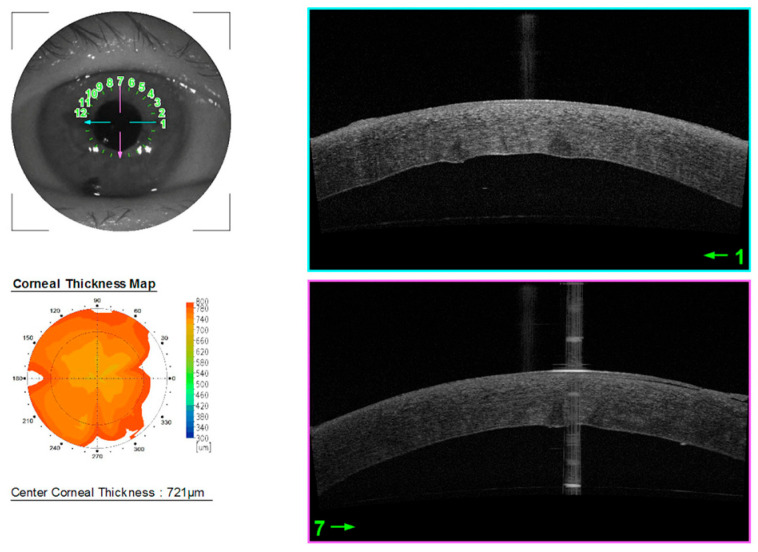
Anterior segment OCT 12 days after surgery.

Indirect microscopy revealed corneal edema. Topical steroid therapy was continued, and the follow-up date was set. Progression of edema to 721 μm was noted 12 days after surgery (Figure 3).

Upside–down orientation of the donor Descemet’s membrane in the anterior chamber was suspected, and the graft was subsequently repositioned. The procedure was performed 33 days after the DMEK.

First, the inner surface of the cornea was stained with trypan blue, and Descemet membrane was stripped and properly positioned under intraoperative OCT.

**Figure 4:**
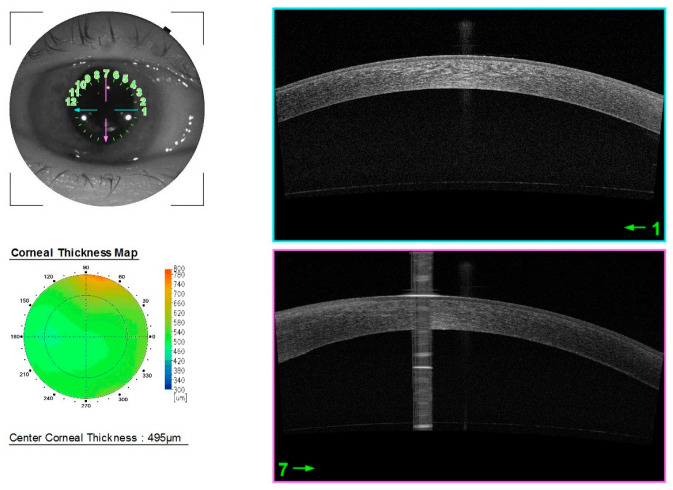
Anterior segment OCT 18 months after surgery.

Anatomical and functional improvements were observed in subsequent controls. At the last follow-up, 18 months after surgery, the CCT was 495 μm, CDVA was 20/40, and CD was 606 cells/mm^2^. The corneas remained completely transparent (Figure 4).

## 3. Discussion

Correct graft orientation is crucial for a successful DMEK outcome. The importance of meticulous surgical techniques and accurate intraoperative decision-making cannot be overstated. As illustrated in our case, misorientation of Descemet membrane can occur even with the use of intraoperative OCT. This highlights the limitations of current imaging modalities, the impact of the learning curve, and the need for standardized orientation confirmation protocols. 

Several factors can affect the accuracy of graft positioning, with the surgeon’s experience being a key factor. Numerous studies have documented a steep learning curve for DMEK, particularly in the first 25–54 cases [11,12]. For instance, Dapena et al. reported significant improvements in graft survival and visual outcomes after the initial 45 procedures [12]. The early experience of our clinic with DMEK may have contributed to the complications described in this case.

Orientation challenges are even greater in eyes with shallow anterior chambers or significant corneal edema, both of which reduce visibility during surgery. Edematous corneas scatter light more diffusely, often producing lower-quality iOCT images despite the high resolution of the technology. Although real-time imaging is an invaluable tool, it is not foolproof, especially when surgical teams are still learning to interpret iOCT results accurately. This highlights the importance of including iOCT-guided graft manipulation as a core component of targeted training programs.

Besides the surgeon’s experience, the properties of the donor tissue play a crucial role during surgery. Donor age affects the biomechanical behavior of Descemet membrane. Grafts from older donors usually scroll more tightly, making them easier to handle but more difficult to unfold. By contrast, tissues from younger donors tend to be more elastic and may scroll less predictably, increasing the risk of accidental inversion [8]. In our case, the donor was 39 years old, which is relatively young for DMEK tissue and may increase handling challenges.

Several methods have been described to determine graft orientation during DMEK. The first one is “S” or “F” marking. The Descemet side of the graft receives a dry ink stamp of an “S” or “F” mark in the peripheral tissue. This pre-stripped, premarked donor tissue is shipped to the surgeon and can be used to verify tissue orientation during trephination, before insertion, after unscrolling the graft in the anterior chamber, and critically before the tissue is lifted into position with a gas bubble. Descemet membrane showed 0.5% cell mortality [7].

Another technique is the Moutsouris sign, which was first described by Dapena et al. [12] in 2011. In this technique, the tip of the cannula is positioned on top of the scroll. If it interacts with a peripheral curl, it turns blue in the case of a right-sided upward graft (Moutsouris sign is positive). If the graft is upside–down, the peripheral curl will not embrace the cannula, and the color of the cannula will remain silver (Moutsouris sign negative) [13].

Rickmann et al. adapted Bhogal’s technique at the end of liquid bubble preparation [14,15]. They prepared a small triangular excision mark that required no equipment other than normal DMEK. This is called shark fin marking and significantly reduces graft turning and re-DMEK rates [15].

In addition, the double-line reflection pattern sign described by Berrospi et al. offers a simple, reproducible method to confirm intraoperative orientation, and could complement or substitute iOCT in settings where the latter is not accessible [9].

Although iOCT has technical limitations, it is especially useful in cases where visibility during surgery is poor, such as in eyes with corneal decompensation or haze. This helps surgeons perform intraocular maneuvers with greater confidence and less reliance on guesswork. Nevertheless, our case demonstrated that iOCT is not foolproof. Limited image resolution or depth, lack of tactile feedback, and limited experience in interpreting scans may have contributed to the initial graft misorientation.

The timing of the graft repositioning in this case was also noteworthy. The revision was performed 33 days after the initial surgery, which is well beyond the commonly recommended 2-week window for correcting upside–down grafts [16]. Delayed intervention is generally associated with increased risk of irreversible endothelial failure. Conventional wisdom suggests that delaying intervention increases the risk of irreversible endothelial failure. However, our patient showed significant corneal clearing and visual improvement (CDVA 20/40) despite a marked drop in endothelial cell density (606 cells/mm^2^ at 18 months). This observation supports the emerging evidence that the corneal endothelium may possess a limited capacity to regenerate or redistribute cells over time, especially if viable donor cells are still present [16].

Nevertheless, the final cell density observed in this case remained below the commonly accepted threshold for long-term graft survival (generally 1000–1500 cells/mm^2^). Whether a patient will develop late endothelial failure remains uncertain, and continued monitoring is essential. This case underscores the importance of the early detection and correction of graft misorientation to preserve endothelial viability and ensure graft longevity.

In the broader context of corneal transplantation, the shift toward ultrathin and selective lamellar procedures such as DMEK represents a significant advancement. However, this evolution increases technical complexity. Although DSAEK offers higher graft survival in less experienced hands, DMEK provides superior optical outcomes and faster visual rehabilitation, provided that the surgery is performed correctly. Therefore, we propose that comprehensive surgeon training, including simulator-based modules and mentoring in high-volume centers, should be prioritized during the rollout of DMEK programs.

Furthermore, future research should focus on improving the reliability of orientation-confirming methods. For example, enhanced real-time imaging tools with automated graft orientation recognition based on reflectivity patterns or machine learning may be of particular interest. The integration of artificial intelligence into intraoperative imaging workflows may, in the future, help reduce operator error and improve consistency across surgical centers.

## Data Availability

The data presented in this study are available within this article.
